# Prenylated phloroglucinols from *Hypericum scruglii*, an endemic species of Sardinia (Italy), as new dual HIV-1 inhibitors effective on HIV-1 replication

**DOI:** 10.1371/journal.pone.0195168

**Published:** 2018-03-30

**Authors:** Cinzia Sanna, Monica Scognamiglio, Antonio Fiorentino, Angela Corona, Vittoria Graziani, Alessia Caredda, Pierluigi Cortis, Mariofilippo Montisci, Elisa Rita Ceresola, Filippo Canducci, Ferruccio Poli, Enzo Tramontano, Francesca Esposito

**Affiliations:** 1 Department of Life and Environmental Sciences, University of Cagliari, Cagliari, Italy; 2 Max Planck Institute for Chemical Ecology—Beutenberg Campus, Jena, Germany; 3 Department of Environmental Biological and Pharmaceutical Sciences and Technologies, University of Campania, Caserta, Italy; 4 Department of Biotechnology and Life Sciences, University of Insubria, Varese, Italy; 5 Laboratory of Microbiology, San Raffaele Hospital, IRCCS, Milan, Italy; 6 Department of Pharmacy and Biotechnology, University of Bologna, Bologna, Italy; Meharry Medical College, UNITED STATES

## Abstract

In a search for new potential multitarget anti-HIV compounds from natural products, we have identified in *Hypericum scruglii*, an endemic and exclusive species of Sardinia (Italy), a potent plant lead. The phytochemical study of the hydroalcoholic extract obtained from its leaves led to the isolation of its most abundant secondary metabolites, belonging to different chemical classes. In particular, three phloroglucinols derivatives were identified, confirming their significance as chemotaxonomic markers of the *Hypericum* genus. Among them, the 3-(13-hydroxygeranyl)-1-(2'-methylbutanoyl)phloroglucinol was reported here for the first time. All six isolated compounds have been evaluated firstly for the inhibition of both Human Immunodeficiency Virus type 1 (HIV-1) Reverse Transcriptase (RT)-associated DNA Polymerase (RDDP) and Ribonuclease H (RNase H) activities, for the inhibition of HIV-1 integrase (IN) in biochemical assays, and also for their effect on viral replication. Among the isolated metabolites, three phloroglucinol derivatives and quercitrin were effective on both RT-associated RDDP and RNase H activities in biochemical assays. The same active compounds affected also HIV-1 IN strand transfer function, suggesting the involvement of the RNase H active site. Furthermore, phloroglucinols compounds, included the newly identified compound, were able to inhibit the HIV-1 replication in cell based assays.

## Introduction

Natural products have played, and will continue to play, a key role in drug discovery. In particular, the diversity of plant-based systems has provided an enormous number of lead compounds in healthcare [1]. Indeed, plant products represent, according to an assessment of FDA on the source of natural products, over one-quarter of all approved new molecular entities [2,3]. However, despite the intensive investigation of plant kingdom, it is estimated that only 6% of the approximately 300,000 species of higher plants have been pharmacologically investigated, and only 15% phytochemically [4]. Therefore, plants should be further investigated because new compounds with original structures and novel modes of action are continuously required. Naturally occurring compounds frequently inspire synthetic medicinal compounds, and they could be chemically modified, based upon their structural and biological properties [5–8]. Their structural modification allows increasing their efficacy and selectivity, improving physicochemical, biochemical and pharmacokinetic properties, removing or reducing side effects.

The therapeutic area of infectious diseases has benefited from abundant scaffold diversity in natural products, able to interact with many specific targets [7]. Significant research and development over the last 25 years into antiviral drug discovery has resulted in the identification of important antiviral drugs [7]. In particular, a number of attempts have been made in the fight against HIV-1 infection and several natural compounds able to inhibit the viral enzymes have been reported [9–17]. However, so far all anti HIV-1 approved drugs were obtained only by chemical synthesis.

HIV-1, the etiological agent of AIDS, still remains a global scourge despite the availability of more than 30 approved anti-AIDS drugs [18]. Although the global scale-up of antiretroviral therapy has contributed to reduce the number of new infections and AIDS-related deaths, about 37 million people were estimated to be infected with HIV in 2016, with 1.8 million of new infections and 1 million of deaths [19]. To date there is no vaccine or cure for HIV infection, and the efficacy of antiretroviral therapy, which combines two or three antiviral agents, targeting different steps of the virus replication cycle, can be compromised by the selection of strains resistant to one or multiple drug classes [20,21] and treatment-associated toxicity [22], requiring the discovery of new antiviral agents with innovative modes of action or targets. In this respect, the identification of one molecule able to inhibit more than one viral function would provide significant advantages, raising the genetic barrier to resistance and increasing the compliance to therapy.

Five different classes of anti-AIDS approved molecules are available for therapy [18] and the majority of them is represented by inhibitors of reverse transcriptase (RT), the enzyme responsible for the conversion of the single-stranded RNA genome into a double-stranded cDNA [23,24]. RT is a multifunctional enzyme with two associated functions [25], DNA polymerase and RNase H activities [26,27], that have been proven to be both essential for viral replication. While the first one is currently the main target for AIDS treatment, the latter is the only HIV enzymatic function not targeted by approved antiviral drugs [26,28,29], although it is a very promising target [30]. Indeed it has been shown that RNase H inactivation lead to non-infectious virions [31] and its selective inhibition completely blocks viral replication [32,33]. RNase H catalytic core is highly conserved among viral species and strains [34,35] and presents high structural homologies with HIV-1 integrase (IN) [24], the enzyme responsible for the integration of the HIV-1 cDNA genome into the host cell chromosome, that takes place through DNA–protein and protein-protein interactions [36]. Among the cellular factors involved in the integration process into the host DNA there is the human lens epitelium-derived growth factor LEDGF/p75 [37], a nuclear protein that promotes IN chromatin tethering by establishing specific interactions between its IN-binding domain and the IN dimer. IN has become an explored target for development of anti HIV treatments [24,38–40], with raltegravir [41,42], elvitegravir [43] and dolutegravir [44] that are IN inhibitors approved for clinical use.

For many years, the drug discovery was based on searching for new compounds or new targets, recently the development of single molecules targeting both viral HIV-1 RT-associated RNase H and RNA-dependent DNA polymerase (RDDP) functions, or RNase H and IN functions (dual inhibitors) has been proposed as an interesting approach [17,26,45–49]. This innovative strategy could offer the possibility to reduce the toxicity associated to the co-administration of several classes of drugs [18,40].

In our ongoing research of new natural compounds as potential scaffolds for developing innovative inhibitors of the HIV-1, we focused on Sardinian endemic flora, in which geographical isolation has selected original metabolic profiles, as documented by several reports [14,50–60].

In particular, in this study we focus on *Hypericum scruglii* Bacchetta, Brullo et Salmeri [61,62], a species exclusive to Sardinia island (Italy). Despite the large number of *Hypericum* species, only *H*. *perforatum* L. has been intensively investigated, both chemically and pharmacologically. Commonly known as St. John’s wort, it is widely used in Europe as a drug for the treatment of mild to moderate depression [63,64]. When compared to *H*. *perforatum*, few studies have been undertaken on the other members of this genus although their recognized pharmacological properties range from wound healing and antiseptics to antiviral, anti-inflammatory, anticancer, antioxidants, antifungal, antimicrobial, cardioprotective and cytotoxic activities[14,51–53,65–69]. Some *Hypericum* species also exhibited anti HIV-1 properties [14,70,71]. This genus is known for the production of a broad spectrum of secondary metabolites, mainly naphthodianthrones (hypericin and pseudohypericin), phloroglucinols (hyperforin and adhyperforin), phenolic acids, flavonoids (hyperoside, rutin or quercitrin), xanthones and essential oils [14,72–75]. Although *H*. *scruglii* was not already characterized in terms of phytochemical composition and biological/pharmacological properties, recently Mandrone et al. [53] have identified from this species shikimic and chlorogenic acids, two known phlorogucinols derivatives, quercitrin, hyperoside and hypericin, even though in a very low content, confirming their chemotaxonomic significance [76]. They have also described the antioxidant and α-glucosidase inhibitory activities.

In the present study, we investigated the ability of the main compounds isolated from leaves of *Hypericum scruglii* to inhibit both HIV-1 RDDP and RNase H activities in biochemical assays. Active compounds were then assayed for their effects on HIV-1 IN activities and to interfere with the HIV-1 life cycle.

## Material and methods

### Plant material

Aerial parts of *H*. *scruglii* were collected at the flowering stage (June 2012) in the site of Sant’Antonio (Jerzu, Sardinia, Italy, 39°45'57.4"N 9°30'41.8"E). The leaves were randomly harvested from 30 individuals of the same population. No flowers, fruits, seeds and roots were collected to avoid damage to the population. The species was botanically identified by C.S. and a voucher specimen (CAG 239/c) was deposited at the General Herbarium of the Department of Life and Environmental Sciences, University of Cagliari. Although *H*. *scruglii* is endemic, it is not protected by local or international regulations. Furthermore, the location where the plant material was harvested is not included in national or local parks or any other natural protected areas. Therefore, no specific permission was required for its collection.

### Chemicals and instruments

Reagents and solvents were purchased from Sigma-Aldrich Chemical Company (St. Louis, MO, USA). The reagents used for expression, purification and biochemical assays were purchased from Microbiol (Sardinia, Italy), Sigma-Aldrich (Milano, Italy) and PerkinElmer (Milano, Italy). The reference compound raltegravir was purchased from ChemScene (Monmouth Junction, United States) while the reference compound RDS1759 was provided from a chemist collaborator Prof. Roberto Di Santo (University of Rome La Sapienza). The isolation of the metabolites was conducted with the aid of distinct chromatographic techniques. The thin-layer chromatographies (TLC) were carried out on plates impregnated silica Merck Kieselgel 60 F_254_ thickness 0.20 mm for analytical purposes and on plates impregnated silica Merck Kieselgel 60 F_254_ thickness 0.5 o 1.0 mm for preparative purposes. The bands were visualized by spraying with the solution H_2_SO_4_-AcOH-H_2_O (1:20:4) and subsequent heating in the stove for 5 min at 120°C. The column chromatographies (CC) were performed using either silica gel Merck Kieselgel 60 (70–240 mesh), Amberlite XAD-4 (20–50 mesh; Fluka) and XAD-7 (20–50 mesh; Fluka), Sephadex LH-20® (Pharmacia) or Bakerbond C8 and C18 as stationary phase.

NMR spectra were recorded at 25°C and 300 MHz for ^1^H and 75 MHz for ^13^C on a Varian NMR spectrometer FT-300. Methanol-d_4_ was used as internal lock.

Correlation spectroscopy (COSY) and double quantum filtered COSY (DQF-COSY) spectra were recorded with gradient enhanced sequence at spectral widths of 3000 Hz in both f2 and f1 domains; the relaxation delays were of 1.0 s. The total correlation spectroscopy (TOCSY) experiments were performed in the phase-sensitive mode with a mixing time of 90 ms. The spectral width was 3000 Hz. For all the homonuclear experiments, the initial matrix of 512 × 512 data points was zero-filled to give a final matrix of 1 k × 1 k points.

Proton-detected heteronuclear correlations were measured. Heteronuclear single-quantum coherence (HSQC) experiments (optimized for ^1^J_(H,C)_ = 140 Hz) were performed in the phase sensitive mode with field gradient; the spectral width was 12000 Hz in f1 (^13^C) and 3000 Hz in f2 (^1^H) and 1.0 s of relaxation delay; the matrix of 1 k × 1 k data points was zero-filled to give a final matrix of 2 k × 2 k points. Heteronuclear 2 bond correlation (H2BC) spectra were obtained with T = 30.0 ms, and a relaxation delay of 1.0 s; the third-order low-pass filter was set for 130 < ^1^J_(C,H)_ < 165 Hz. Heteronuclear multiple bond coherence (HMBC) experiment (optimized for ^n^J_(H,C)_ = 8 Hz) was performed in the absolute value mode with field gradient; typically, ^1^H–^13^C gHMBC were acquired with spectral width of 18000 Hz in f1 (^13^C) and 3000 Hz in f2 (^1^H) and 1.0 s of relaxation delay; the matrix of 1 k × 1 k data points was zero-filled to give a final matrix of 4 k × 4 k points. Constant time inverse-detection gradient accordion rescaled heteronuclear multiple bond correlation spectroscopy (CIGAR–HMBC) spectra (8> ^n^J_(H,C)_ >5) were acquired with the same spectral width used for HMBC. Heteronuclear single-quantum coherence—total correlation spectroscopy (HSQC-TOCSY) experiments were optimized for ^n^J_(H,C)_ = 8 Hz, with a mixing time of 90 ms.

LC-MS analysis was carried out on an Alliance 2695 separation module equipped with a column heater and a sample chiller. The liquid chromatography system was coupled to a Waters 2487 dual wavelength UV detector and to a Quattro Micro™ triple quadrupole mass spectrometer (Waters/Micromass, Manchester, UK).

Recombinant proteins were purified using the chromatography system Biological LP (Biorad). Biochemical assays were measured using the multiplate reader Victor 3 (Perkin Elmer).

### Extraction and isolation of active compounds

Plant material (70.0 g) was extracted by sonication with a solution of H_2_O:MeOH (1:1), immersed in an ultrasonic bath (Elma®Transonicdigitals) for 40 min. Subsequently, samples were filtered and the obtained crude extract was solubilized in H_2_O and then subjected to liquid-liquid extraction using ethyl acetate (EtOAc) as extracting solvent. The aqueous fraction was chromatographed on Amberlite XAD-4 and XAD-7, eluting first with water and then with methanol. The organic fraction (10.0 g) was chromatographed on silica gel (SiO_2_CC) using CHCl_3_/MeOH solutions as eluent to afford 13 fractions. Fraction 1, eluted in chloroform, was chromatographed through Sephadex LH-20, using *n*-hexane/CHCl_3_/MeOH (2:1:1) as eluent solution; four fractions (A-D) were obtained. Fraction A was chromatographed by semi preparative TLC (0.5 mm) using CHCl_3_/MeOH (1:19) as eluent. The compounds **3** was obtained. From fraction B compounds **1** and **2** were isolated. Fraction 2, eluted with CHCl_3_/MeOH (95:5), chromatographed by RP-18 CC using decreasing polarity H_2_O/MeOH solutions, led to compounds **4** and **5**.

Fraction 3 eluted with CHCl_3_/MeOH (9:1), was chromatographed by RP-18 CC using decreasing polarity H_2_O/MeOH solutions, obtaining the compound **6**.

### Expression and purification of recombinant HIV-1 RT, IN and LEDGF

His-tagged p66/p51 HIV-1 RT was expressed in *Escherichia coli* strain M15 as previously described [77]. Full-length IN and LEDGF proteins were expressed in *E*. *coli* BL21 (DE3) [15,78].

### RNase H polymerase-independent cleavage assay

The HIV-1 RT-associated RNase H activity was measured in 100 μL reaction volume containing 50 mM Tris–HCl, pH 7.8; 6 mM MgCl_2_; 1 mM dithiothreitol (DTT); 80 mM KCl; hybrid RNA/DNA (5′-GTTTTCTTTTCCCCCCTGAC-3′-fluorescein, 5′-CAAAAGAAAAGGGGGGACUG-3′-Dabcyl); and 3.8 nM RT. The reaction mixture was incubated for 1 h at 37°C, the reaction was stopped by the addition of EDTA, and products were measured with a Victor 3 (Perkin) at 490/528 nm [35].

### RDDP assay

The HIV-1 RT-associated RDDP activity was measured using the Enz-Check Reverse Transcriptase Assay Kit (Life technologies, Carls- bad, California, USA), as previously described [47]. The Yonetani-Theorell analysis was performed as previously reported [79].

### Homogeneous time resolved fluorescence (HTRF) LEDGF dependent assay

The IN LEDGF/p75 dependent assay allowed to measure the inhibition of the 3’processing and strand transfer IN reactions in the presence of recombinant LEDGF/p75 protein, as previously described [80]. Briefly, 50 nM IN was preincubated with increasing concentration of compounds for 1 hour at room temperature in reaction buffer containing 20 mM HEPES pH 7.5, 1 mM DTT, 1% Glycerol, 20 mM MgCl_2_, 0.05% Brij-35 and 0.1 mg/ml BSA. To this mixture, 9 nM DNA donor substrate (5’-ACAGGCCTAGCACGCGTCG-Biotin-3’ annealed with 5’-CGACGCGTGGTAGGCCTGT-Biotin3’) and 50 nM DNA acceptor substrate (5’-Cy5-ATGTGGAAAATCTCTAGCAGT-3’ annealed with 5’-Cy5- TGAGCTCGAGATTTTCCACAT-3’) and 50 nM LEDGF/p75 protein were added and incubated at 37°C for 90 minutes. After the incubation, 4 nM of Europium-Streptavidine were added at the reaction mixture and the HTRF signal was recorded using a Perkin Elmer Victor 3 plate reader using a 314 nm for excitation wavelength and 668 and 620 nm for the wavelength of the acceptor and the donor substrates emission, respectively.

### Antiviral activity and cell toxicity. Phenotypic analyses with fully replicating recombinant HIV-1 strain

The human TZM-bl indicator cell line was obtained from the American Type Culture Collection (Manassas, VA) and maintained at 37°C and 5% CO_2_ in Dulbecco's modified Eagle's medium (DMEM) containing 10% fetal bovine serum, 50 μg/mLpenicillin, and 50 μg/mL streptomycin. The HIV-1 virus NL(AD8) was titrated as follows: serial 5-fold dilutions of the virus were made in quadruplicate wells in 96-well culture plates, in a total volume of 100 μL of growth medium, for a total of 8 dilution steps. Freshly trypsinized cells (20,000 cells in 100 μL of growth medium containing 75 μg/mL DEAE-dextran) were added to each well, and the plates were incubated at 37°C in a humidified 5% CO_2_-95% air environment. After 48 h of incubation, the medium was removed and viral infection was quantified using a β-galactosidase (CPRG) assay (Roche). Twenty thousand TZM-bl cells/well were seeded in 96-well plates in complete DMEM supplemented with 30 μg/mL DEAE-dextran (Sigma-Aldrich). Three hundred times the 50% tissue culture infective dose (TCID50)/mL of HIV AD8 strain and seven serial dilutions (range, 40.000 nM to 625 nM) of each compound were added to the cells, as previously described [81,82]. Vehicle (0.1% dimethyl sulfoxide [DMSO])-treated cells served as a negative control. A CCR5 inhibitor (maraviroc) and an integrase inhibitor (dolutegravir) were used as positive-control drugs. The TZM-bl indicator cell line has an integrated copy of the β-galactosidase gene under control of the HIV-1 promoter. Β-Galactosidase expression, measured by use of a chlorophenol red/β-D-galactopyranoside (CPRG) assay (Roche) in cell lysates 48 h post-infection was used as a marker of HIV infection. The inhibitory curves were fitted by nonlinear regression, allowing for the calculation of the 50% effective concentration (EC_50_) using the Prism software. To evaluate the cell toxicity of the compounds, the metabolic XTT [2,3-bis-(2-methoxy-4-nitro-5-sulfophenyl)-2H-tetrazolium-5-carboxanilide] test (Sigma-Aldrich) was performed according to the manufacturer's instructions.

### Time of addition

In TOA experiment it is of fundamental importance that only one replication cycle has been completed to avoid confounding effects derived from unwashed viruses after 1 hour of infection. For this reason, an env-pseudotyped virus (REJO4541 clone 67) was used. Time of addition (TOA) experiment was performed as previously described with minor modifications [83]. 40000 TZM-bl cells/well in a 96-multiwell plate were infected with 1500 X the TCID50 per mL of the env-pseudotyped HIV-1 virus in complete medium supplemented with DEAE-dextran (Sigma–Aldrich, 30 mg/mL). Virus was incubated with cells for 1 h at 4°C, and unbound virus was subsequently removed by extensive and repeated washing with phosphate-buffered saline (PBS) to synchronize the replication. For the next 7 h, antiretroviral compounds inhibiting distinct viral replication steps (maraviroc, lamivudine, dolutegravir) and compound 3 were added at the following time points: at time 0 and after 60, 120, 180, 240, 300, 360 and 420 min. To ensure complete inhibition of viral replication we used a 40-fold EC_50_ concentration as previously evaluated for each compound on TZM-bl cells [maraviroc (0.7 mM), lamivudine (5 mM), dolutegravir (1 mM), and compound 3 (10 mM)]. After 2 days, viral infection was quantified using a CPRG assay (Roche) and was then normalized to untreated control cells.

## Results and discussion

### Phytochemical profile

**Fig 1:**
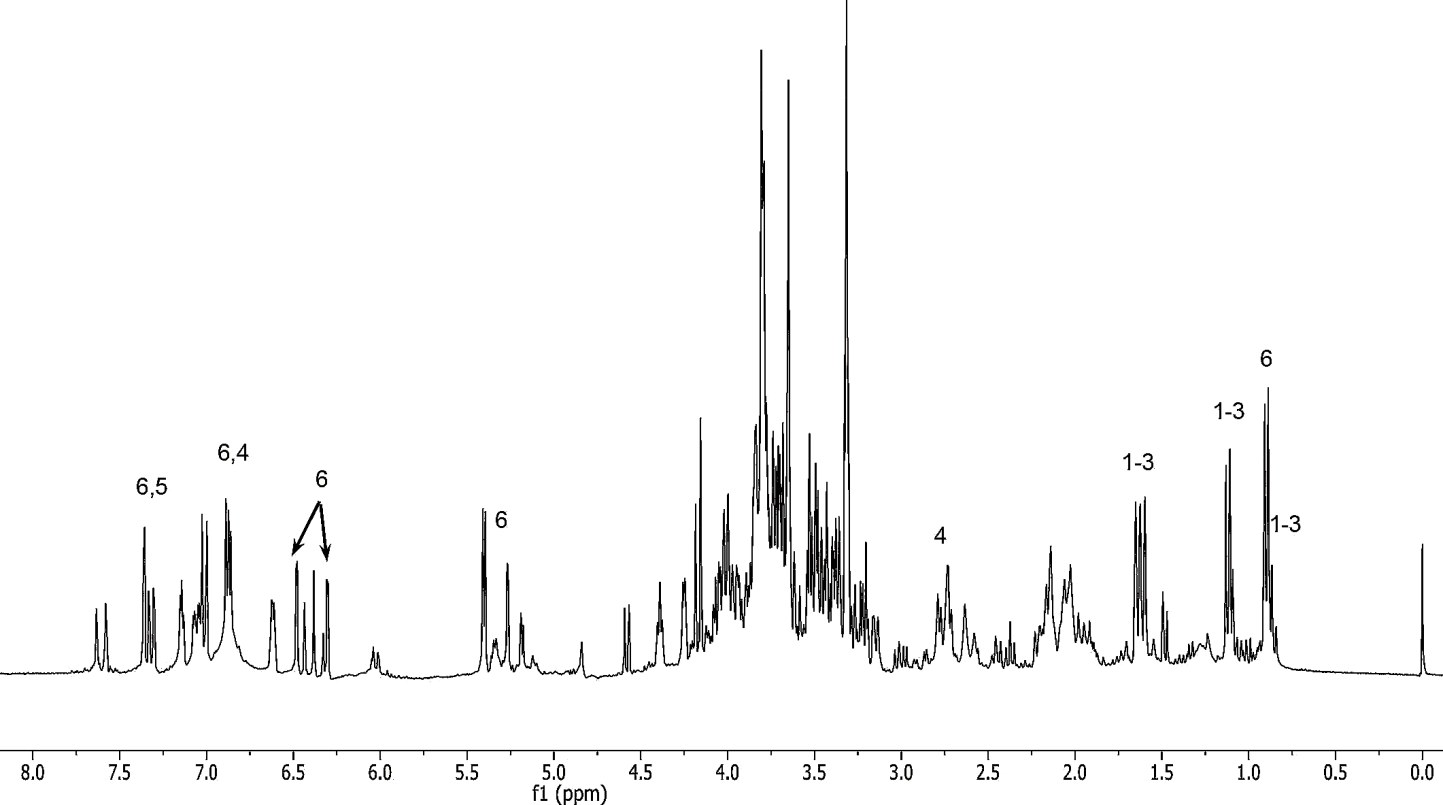
^1^H NMR spectra of MeOH/H_2_O extract of *H*. *scruglii*. Numbers indicate the diagnostic signals of the isolated secondary metabolites 1–6.

Based on previous observations [53], a phytochemical study was undertaken in order to isolate the most abundant compounds in the hydroalcoholic extract obtained from leaves of *H*. *scruglii* (Fig 1). These compounds belong to different classes and, among them, are three phloroglucinol derivatives (compounds **1**–**3**). Three compounds (**3**–**5**) were reported and isolated for the first time from this species and compound **3** was reported here for the first time.

**Fig 2:**
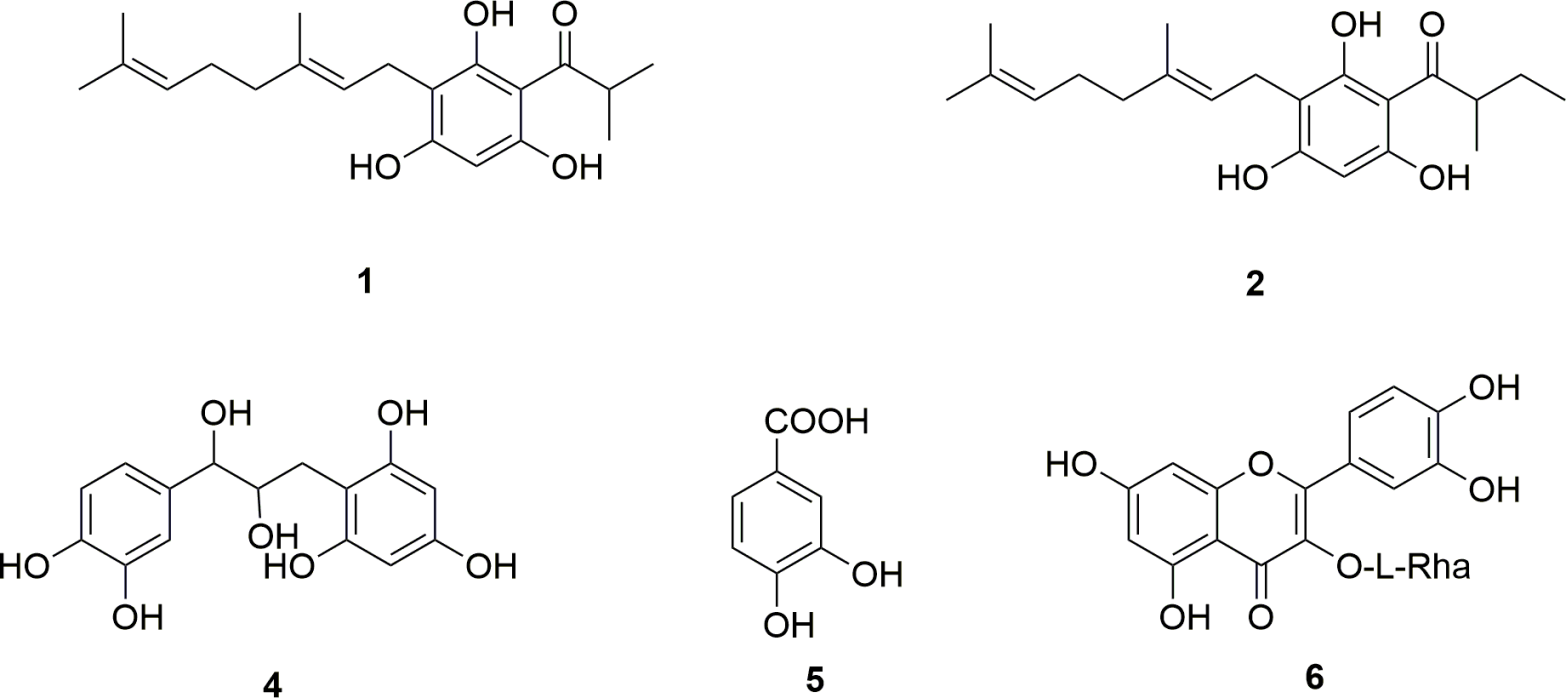
Chemical structures of known metabolites isolated from *H*. *scruglii*.

The phytoconstituents isolated were 3-geranyl-1-(2'-methylbutanoyl)phloroglucinol (**1**), 3-geranyl-1-(2'-methylpropanoyl)phloroglucinol (**2**), 3-(13-hydroxygeranyl)-1-(2'-methylbutanoyl)phloroglucinol (**3**), 1,3,5-benzentriol 2-[(2S,3R)-3-(3,4-dihydroxylphenyl)-2,3-dihydroxylpropyl] (**4**), 3,4-dihydroxybenzoic acid (**5**) and quercitrin (**6**) (Fig 2).

Compounds **1**, **2**, and **3** belong to the class of phloroglucinols. The NMR data for compound **1** and **2** were in agreement with 3-geranyl-1-(2'-methylbutanoyl)-phloroglucinol and 3-geranyl-1-(2'-methylpropanoyl)-phloroglucinol, respectively, previously reported from other *Hypericum* species [71,84] and also identified in the extract of *Hypericum scruglii* through NMR-based metabolomics[53].

**Fig 3:**
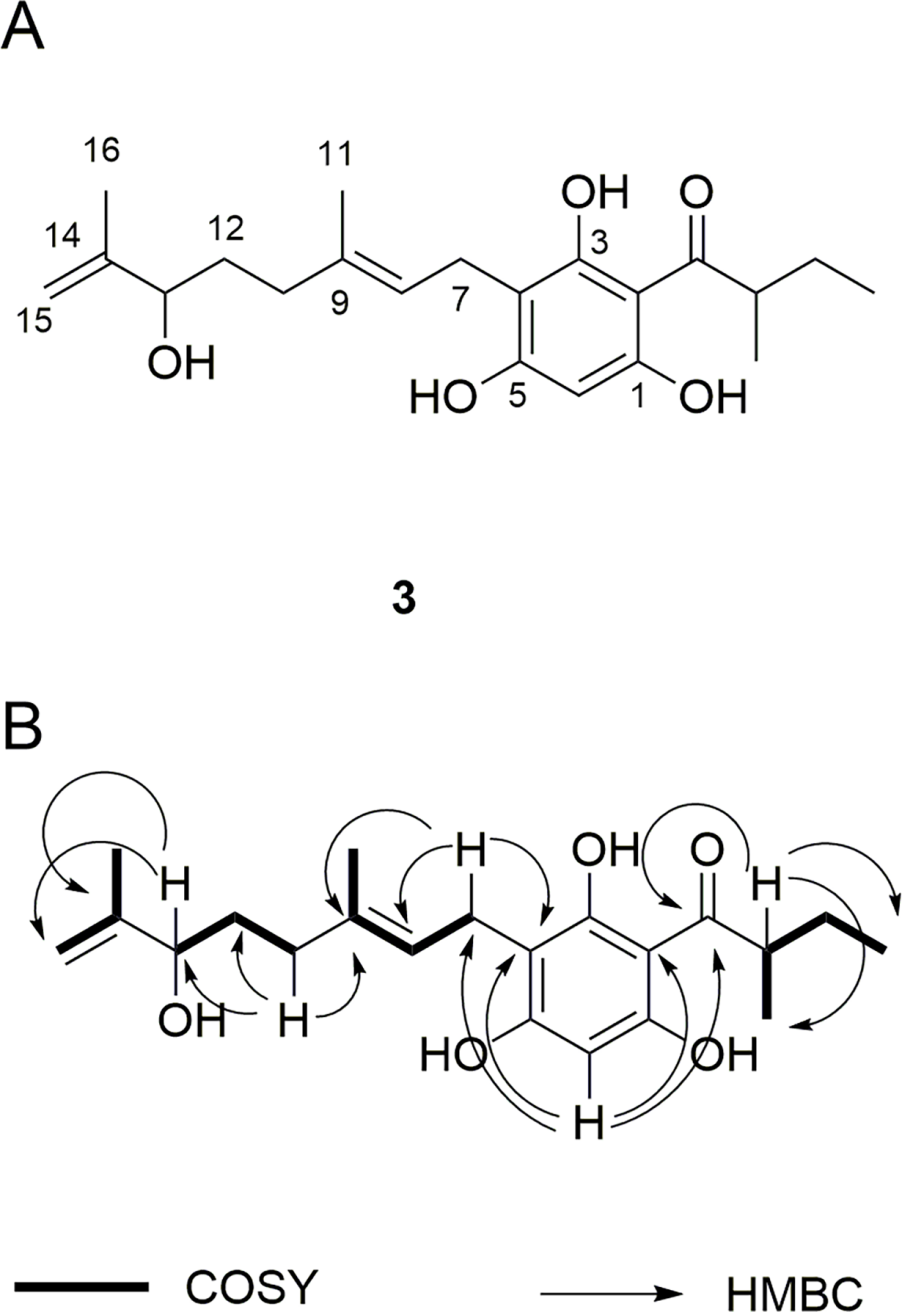
A: Chemical structure of the novel phloroglucinol 3 from *H*. *scruglii*; B: diagnostic 2D NMR correlations.

Compound **3** (Fig 3) has been identified as a new metabolite on the basis of its spectroscopic features (Table 1). Its ^13^C-NMR spectrum shows 21 signals identified through ^13^C and HSQC experiments as 4 methyls, 5 methylenes, 4 methines and 8 quaternary carbons, one of them, at δ 210.1, attributable to a carbonyl group.

**Table 1:** 1D and 2D NMR data of compound 3 in CD_3_OD.

n^o^	δ ^1^H	J (Hz)	DQ-COSY	δ ^13^C	HSQC	CIGAR-HMBC	HSQC-TOCSY
			H→H			H→C (^n^J)	H→C
1				161.1	C		
2				105.1	C		
3				165.4	C		
4				108.0	C	4(^2^J), 5(^3^J), 8(^2^J), 9(^3^J)	
5				163.5	C		
6	5.88	s		94.8	CH	1(^2^J), 2(^3^J), 4(^3^J), 5(^2^J), 7(^4^J), 1’(^4^J)	
7	3.19	d (6.9)	8	21.9	CH_2_	2(^3^J), 3(^3^J), 4(^2^J), 5(^3^J), 8(^2^J), 9(^3^J)	7, 8, 11(*lr*)
8	5.19	t (6.0)	7, 11(*lr*)	125.5	CH	7(^2^J), 10(^3^J)	7, 8, 11(*lr*)
9				135.1	C		
10	2.20	m	12	29.1	CH_2_	8(^3^J), 9(^2^J), 11(^3^J), 12(^2^J), 13(^3^J)	10, 12, 13
11	1.65	s	8(*lr*)	23.7	CH_3_	8(^3^J), 9(^2^J), 10(^3^J)	
12	1.62	m	10, 13	34.4	CH_2_	13(^2^J), 14(^3^J)	10, 12, 13
13	4.03	t (6.6)	12	77.0	CH	10(^3^J), 12(^2^J), 14(^2^J), 15(^3^J), 16(^3^J)	10, 12, 13
14				149.1	C		
15a	4.95	s	15b, 16(*lr*)	111.4	CH_2_	13(^3^J), 14(^2^J)	14, 16
15b	4.83	s	15a, 16(*lr*)	111.4	CH_2_	13(^3^J), 14(^2^J), 16(^3^J)	14, 16
16	1.75	s	15b(*lr*), 15a(*lr*)	17.7	CH_3_	13(^3^J), 14(^3^J), 15(^2^J)	14, 16
1’				211.7	C		
2’	3.87	tq (6.6)	3’, 5’	46.7	CH	1’(^2^J), 3’(^2^J), 4’(^3^J), 5’(^2^J)	2’, 3’, 4’, 5’
3’a	1.34	m	2’, 4’	28.1	CH_2_	1’(^3^J), 2’(^3^J), 4’(^2^J), 5’(^3^J)	2’, 3’, 4’, 5’
3’b	1.80	ov	2’, 4’	28.1	CH_2_	1’(^3^J), 2’(^3^J), 4’(^2^J), 5’(^3^J)	2’, 3’, 4’, 5’
4’	0.89	t (7.2)	3’	12.4	CH_3_	2’(^3^J), 3’(^2^J)	2’, 3’, 4’, 5’
5’	1.10	d (6.6)	2’	17.3	CH_3_	2’(^2^J), 3’(^3^J)	2’, 3’, 4’, 5’

d = doublet, m = multiplet, ov = overlapped, s = singlet, t = triplet, tq = triplet of quartet; *lr* = long range.

In the aromatic region of the ^1^H-NMR spectrum, a singlet signal at δ 5.88 is observed, which correlates, in the HSQC spectrum, with the carbon at δ 93.5 and, in the CIGAR-HMBC spectrum, with the C-1, C-2, C-4 and C-5 at δ 161.1, 105.1, 108.0, 163.5, respectively. The methine proton at δ 3.87, linked to carbon C-2' at δ 46.7, correlated in the CIGAR-HMBC experiment, with the carbonyl carbon and with the C-5’ methyls at δ 17.3 and δ 12.4, and with the methylene diasterotopic protons at δ 1.34 and δ 1.80 bonded to carbon C-3' δ 28.1. These correlations were in good agreement with the presence of a 2-methylbutanoyl group on the aromatic ring. The C-4 carbon of the aromatic ring at δ 108.0 correlates, in the CIGAR-HMBC, with the H-7 methylene doublet at δ 3.19. This proton is directly bonded to a carbon at δ 23.7, and it shows long range correlations with the aromatic ring and with the C-8 and C-9 olefinic carbon at δ 125.5 and 135.1, respectively. Furthermore, the H-8 olefinic proton at δ 5.19 correlates with the methyl group linked to the C-9 and the C-10 methylene carbon at δ 29.1, bonded to the protons at δ 2.20.

The H-12 protons at δ 1.62, directly linked to the methylene carbon at δ 23.7, correlates with the C-13 and C-14 carbons at δ 77.0 and 149.1, respectively. This latter presents correlations with the methyl protons at δ 1.75, bonded to the carbon at δ 17.7, and with the olefinic protons at δ 4.83 and 4.95 both correlated in the HSQC experiment to the carbon at δ 111.4.

The correlations observed in the CIGAR-HMBC, H2BC and HSQC-TOCSY spectra suggested the structure of compound **3** (Fig 3). These data allowed the identification of compound 3 as 3-(13-hydroxygeranyl)-1-(2'-methylbutanoyl)phloroglucinol.

Compound **4** was identified as 1,3,5-benzentriol 2-[(2S,3R)-3-(3,4-dihydroxylphenyl)-2,3-dihydroxylpropyl], named filiferol, a chalconoid analogue, isolated for the first time from leaves of *Washingtonia filifera* [85]. It is based on a flavonol structure with the reduction of the common flavonoid keto group to give an unprecedented methylene carbon on the three carbon chain. To our knowledge, this is the first report of filiferol from *Hypericum* genus.

3,4-dihydroxybenzoic acid (**5)**, is a phenolic acid widely distributed in nature [86]. This compound is one of the biologically active components of some medicinal plants, including *Hypericum perforatum* L. [87]. It is been defined by the presence of characteristic signals in the ^1^H-NMR spectrum.

Finally, compound **6**, was identified as quercitrin, a quercetin glycoside. It was already reported for *H*. *scruglii* [53] and it is commonly present in the genus *Hypericum* [75,76,88–95].

### Effects of *H*. *scruglii* chemical components on both HIV-1 RT-associated functions

Biological activities of *H*. *scruglii* were previously investigated reporting its antioxidant and α-glucosidase activity [53]. However, up to date, no information on its anti HIV-1 properties was available. Given the promising results obtained from *Hypericum hircinum* L. components on both HIV-1 RT associated functions [14], with the objective to identify new metabolites able to inhibit both HIV-1 RT-associated functions from the *Hypericum* genus, the most abundant compounds (**1–6**) isolated from *H*. *scruglii* have been tested on both RDDP and RNase H activities in biochemical assays, using the RDDP selective non-nucleoside RT inhibitor (NNRTI) Efavirenz and the RNase H selective diketo acid (DKA) derivative RDS1759 [33] as controls (Table 2). In accordance to other reports on naturally occurring phloroglucinol compounds that have shown a broad range of biological activities including anti-HIV activity [71,96–99], our results showed that 3-geranyl-1-(2'-methylbutanoyl)phloroglucinol (**1**), 3-geranyl-1-(2'-methylpropanoyl)phloroglucinol (**2**), 3-(13-hydroxygeranyl)-1-(2'-methylbutanoyl)phloroglucinol (**3**) inhibited both HIV-1 RT-associated activities with IC_50_ values around 4.1–25.5 μM range (Table 1). Interestingly, the small differences in the lateral chains of **1**, **2** and **3** do not affect the potency of inhibition towards the two viral functions. Also quercitrin (**6**) showed to be active in the low micromolar range against both RT-associated functions. Quercitrin (**6**) is indeed a glycoside of quercetin, a flavonoid which is known to be a potent inhibitor of both functions of HIV-1 RT [14,100]. Differently, 1,3,5-benzentriol 2-[(2S,3R)-3-(3,4-dihydroxylphenyl)-2,3-dihydroxylpropyl], known as filiferol (**4),** exhibited a weak inhibition of both HIV-1 RT-associated RNase H and RDDP functions, and 3,4-dihydroxybenzoic acid, namely protocatechuic acid (**5)**, was found inactive at the maximum concentration tested (100 μM), similarly to what already found for the structurally-related shikimic acid [14].

**Table 2:** Effects of compounds isolated from *Hypericum scruglii* on the HIV-1 RT-associated activities and IN activities in presence of LEDGF/p75.

Compound	^a^HIV-1 RT	^b^HIV-1 RT	^c^HIV-1 IN LEDGF
	RNase H IC_50_ (μM)	RDDP IC_50_ (μM)	dependent IC_50_ (μM)
**1**	4.3 ± 0.4	25.5 ± 8.8	7.3 ± 0.3
**2**	4.1 ± 0.1	12.3 ± 2.5	7.4 ± 0.4
**3**	9.1 ± 0.5	19.7 ± 3.5	13.0 ± 1.0
**4**	93 ± 7	92 ± 10	6.4 ± 0.7
**5**	> 100 (100%)^d^	> 100 (85%)^d^	>100 (97%)^d^
**6**	6.3 ± 1.0	9.7 ± 1.4	1.6 ± 0.16
**RDS 1759**	7.3 ± 0.1		
**Efavirenz**		0.012 ± 0.003	
**Raltegravir**			0.058 ± 0.01

^a^Compound concentration required to inhibit the HIV-1 RNase H activity by 50%.

^b^Compound concentration required to inhibit the HIV-1 RDDP activity by 50%.

^c^Compound concentration required to inhibit the HIV-1 IN catalytic activities, in the presence of LEDGF, by 50%.

^d^Percentage of control activity in the presence of 100 μM concentration of compound.

### Evaluation of the effects *H*. *scruglii* chemical components on HIV-1 IN activity

Since HIV-1 RNase H and IN domains have striking similarities, in order to evaluate if the compounds able to inhibit HIV-1 RNase H function could act through a multitarget profile, we investigated them also on IN catalytic activities. It is well known, in fact, that compounds capable to inhibit the HIV-1 RNase H activity may also affect the HIV-1 IN activity [15,17,39,45,46,80]. Hence, we evaluated their ability to inhibit the HIV-1 IN strand transfer reaction in the presence of the LEDGF/p75 cellular cofactor, using Raltegravir as positive control (Table 2).

Results showed that 3-geranyl-1-(2'-methylbutanoyl)phloroglucinol (**1**), 3-geranyl-1-(2'-methylpropanoyl)phloroglucinol (**2**), 3-(13-hydroxygeranyl)-1-(2'-methylbutanoyl)phloroglucinol (**3**) inhibited HIV-1 IN activities in presence of LEDGF/p75 with IC_50_ values in the 7.3–13μM range. Also in this case, results showed that the small differences in the lateral chains of **1**, **2** and **3** do not affect the potency of inhibition on these enzymatic functions. It is worth noting that these compounds were able to inhibit in similar concentration both RT-associated RNase H activity and IN strand transfer function, while they were found to be less active on RT-associated RDDP activity. Differently, quercitrin (**6**) inhibited HIV-1 IN in the low micromolar range, resulting 4-fold more active on HIV-1 IN activity with respect to HIV-1 RNase H function. The HIV-1 Integrase (IN) inhibition of its aglycon quercetin has been already reported in *in vitro* assay [101]. Worth to note, the filiferol (**4**) that weakly inhibited both HIV-1 RT-associated RNase H and RDDP functions, were able to inhibit the HIV-1 IN activities in presence of LEDGF/p75 with an IC_50_ value of 6.4 μM, showing a selectivity for this viral enzyme. 3,4-dihydroxybenzoic acid (**5**), already found inactive on both RT-associated activities, was not active also on HIV-1 IN inhibition.

### Inhibition of HIV-1 in cell culture and characterization of the mechanism of action of bioactive compounds in cell-based assays

Given that compounds **1**, **2**, **3**, **4** and **6** were able to inhibit both the HIV-1 RT and IN functions in biochemical assays, we wanted to evaluate their effect on the HIV-1 replication.

**Fig 4:**
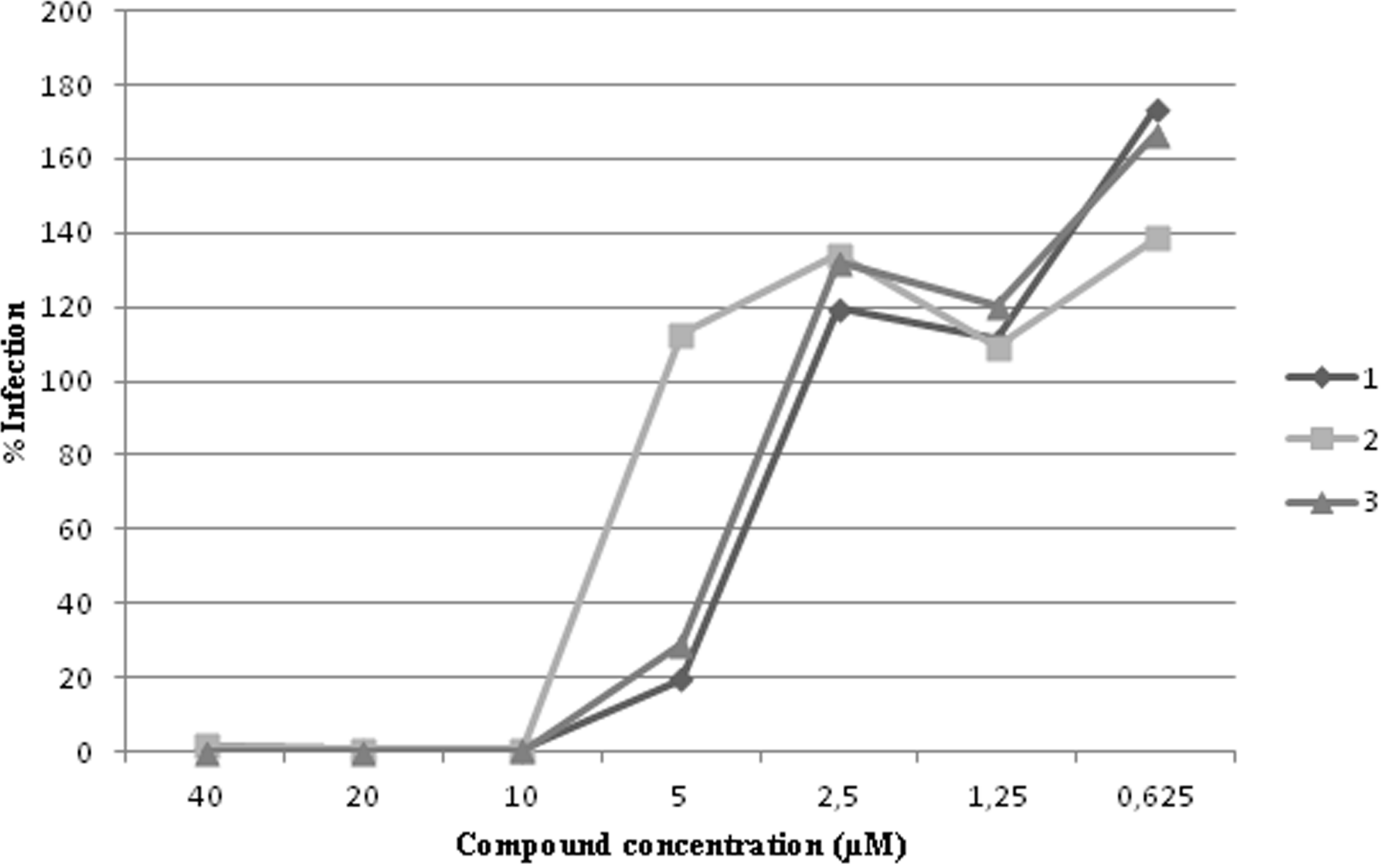
Antiviral activity of compounds 1, 2 and 3 on HIV AD8 laboratory strain in TZM-bl cells. Cells were infected with 300 TCID50/mL and treated with compounds isolated from *H*. *scruglii* at seven different concentration. EC_50_ values ranged from 3.5 to 8 **μ**M. Only active compounds were shown.

**Fig 5:**
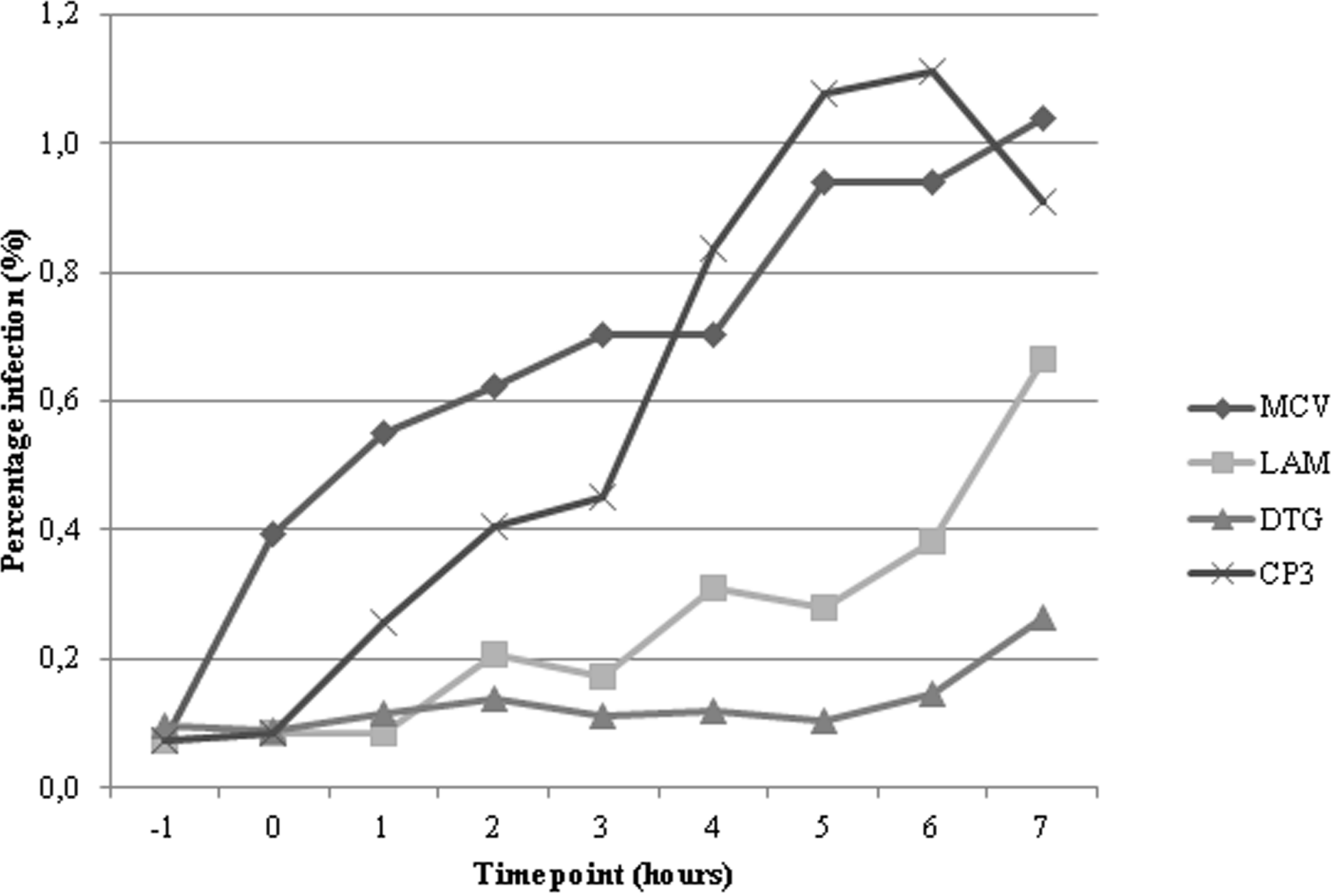
Time-of-addition assay. The target of the antiviral compound **3** (Cp3) was identified by comparing its activity in the time scale to those of reference drugs: Maraviroc (MCV, entry inhibitor), Lamivudine (LAM, RT inhibitor), Dolutegravir (DTG, IN inhibitor). Cp3 was ineffective once the virus retrotranscribed its genome.

Results showed that compounds **1**, **2** and **3** significantly inhibited HIV-1 replication with EC_50_ values in the 3.5–8 μM range (Fig 4), in accordance with the range of IC_50_ values showed against the three viral enzymatic functions in biochemical assays, showing no cytotoxic effect up to the highest tested concentration in cells (CC_50_ > 50 μM) (Table 3). Quercitrin, even if it was able of inhibiting both HIV-1 RT-associated activities and IN functions in biochemical assay, did not exert any effect on HIV-1 replication at the highest tested concentration (Table 3), similarly to what reported for its aglycon quercetin [102]. Since compounds **1**, **2** and **3** were active on both HIV-1 RT and IN in biochemical assays, we asked which was the viral process targeted by bioactive compounds and, hence, a time-of-addition experiment on the most promising molecule was carried out. This experiment determines how long the addition of an anti-HIV compound can be postponed within the viral replication cycle before losing its antiviral activity. Reference compounds with a known mode of action such as Maraviroc, Lamivudine and Dolutegravir were included. As shown in Fig 5, the compound **3**, similarly to lamivudine, a RT inhibitor, lost its activity if added after 4–5 hour post-infection. This timing is compatible with an anti-RT activity, exerted on both RNase H and RDDP functions, but not with IN inhibition [103]. These data demonstrate that compound **3** exerts its anti-HIV activity targeting the RT functions, while the anti-IN activity, exhibited only in enzymatic assays, is not significantly involved in the inhibition of viral replication. A number of compounds were reported to have dual RNase H/IN inhibitory activity in vitro but were more selective for IN and, indeed, they were shown to inhibit the IN in cell-based experiments [104]. Very few compounds [32,33] were reported to be more selective for RNase H versus IN in vitro and were shown to inhibit the RT in cell-based experiments. Hence, it is possible that a dual inhibitory activity displayed in enzymatic assays, is not supported by cell-based results, but at our knowledge, no much data are available on such discrepancies on compounds that were shown to have equal potency of inhibition in vitro on the two enzymes. Therefore, further studies should be performed to elucidate this specific behavior and to obtain derivatives of compound **3** that may be active on viral replication targeting both viral enzymes.

**Table 3:** Effects of compounds isolated from *Hypericum scruglii* on the HIV-1 replication.

Compounds	^a^EC_50_ (μM)	^b^CC_50_(μM)
**1**	3.5	>50
**2**	8	>50
**3**	3.5	>50
**4**	>40	
**5**	>40	
**6**	>40	
**Maraviroc**	0.07	>20

^a^Compound concentration required to inhibit HIV-1 (AD8) replication in TZM-bl cells by 50%.

^b^Compound concentration required to inhibit TZM-bl cell viability by 50%.

## Conclusions

Searching for new potential multitarget anti-HIV active compounds form Sardinian endemic flora, we successfully identified in *Hypericum scruglii* some chemical components able to inhibit both HIV-1 RT-associated and IN activities in the low micromolar range. Among the bioactive compounds, two known phloroglucinol derivatives, compounds **1** and **2**, and a newly identified acylphloroglucinol, compound **3**, were also able to inhibit the virus replication in cell-based assays. Mode of action studies demonstrated that these compounds were active also in cell cultures and the timing of inhibition was compatible with an action on the HIV RT enzyme.

Hence, bioactive compounds isolated from *H*. *scruglii*, represent new attractive scaffolds for the development of new dual inhibitors that deserve further investigations by means of chemical modification, in search of new dual derivatives active on both HIV-1 RT and IN.
